# Hyaluronic Acid Could Cut Cancer Risk, if We Can Get around Its Cons

**DOI:** 10.1021/acscentsci.6c01378

**Published:** 2026-08-21

**Authors:** Rachel Brazil

## Abstract

The antiaging molecule, when broken down, can drive inflammation. Drugmakers are trying to reap its benefits while minimizing harm.

## Graphical abstract

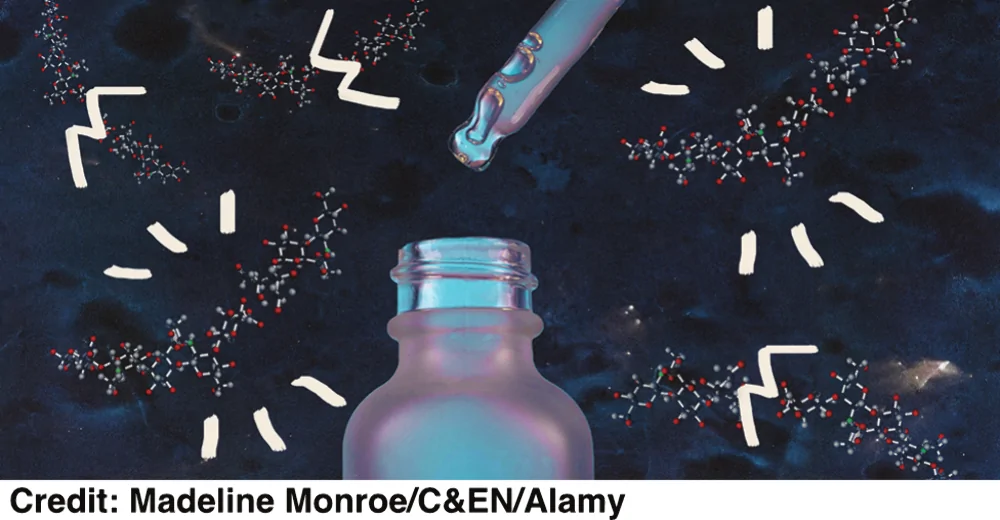

The naked mole rat is a rodent, but it is “nothing like a mouse,” says biologist Vera Gorbunova from the University of Rochester Aging Research Center. These tiny hairless animals, native to eastern Africa, live in colonies of hundreds and can survive without drinking liquid water.

**Figure:**
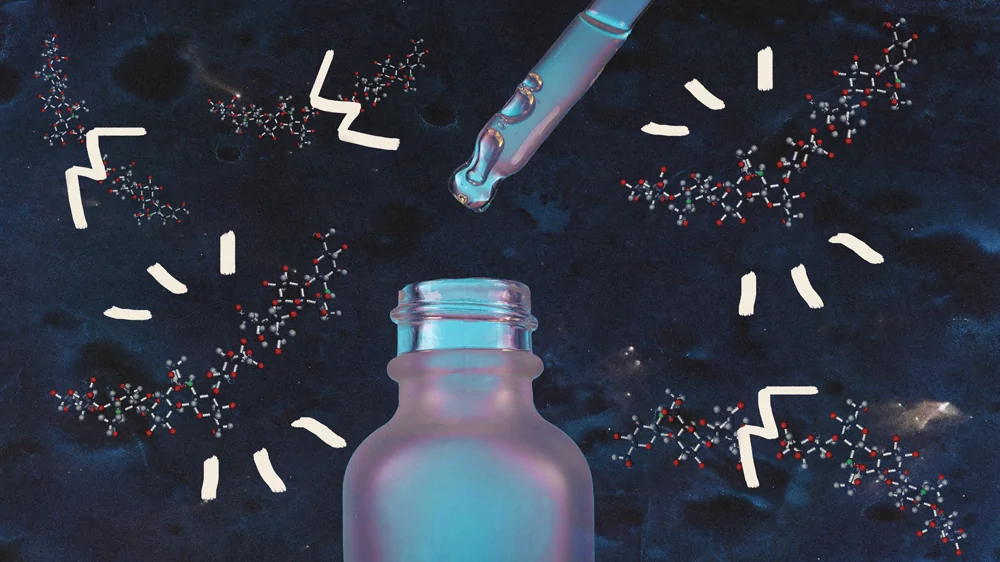
Hyaluronic acid is a well-known ingredient in antiaging skin products. It’s also naturally occurring, with the human body producing about 5 g per day. Researchers are gaining a better understanding of the molecule’s biochemistry. Credit: Madeline Monroe/C&EN/Alamy.

And of particular interest to Gorbunova is their longevity—they can live for 40 years—and their resistance to cancer and other diseases.

In 2013, Gorbunova showed that their exceptional lifespan is due to long chains of sugars that pervade the spaces between their cells. These molecular chains, called extremely high-molecular-mass hyaluronic acid (HA), provide the animal with an elastic skin, evolved for its tunnelling lifestyle. The viscous polymer is also able to retain water, making it ideal for plumping up skin wrinkles, and hence bacterially fermented HA is widely used in cosmetics.

But the protection afforded by the longest HA chains is only half the story. “Depending on the length, it has different biological effects, and that is what makes it so fascinating and also challenging to study,” says Gorbunova.

Research over the past decade has shown that HA is caught in a balancing act. The heaviest, longest form of the molecule protects tissues and lowers inflammation, while shorter fragments act in the opposite way, driving disease and aging.

Drug start-ups and scientists are now aiming to prevent damage from these short HA fragments. Some approaches involve blocking the enzymes that break HA down, and more-radical methods involve reducing the synthesis of HA altogether. Regardless of the technique, researchers are trying to strike a delicate balance; can they stave off the downsides of HA fragmentation without compromising the molecule’s benefits?

## Short and long chains

HA, also known as hyaluronan, is made from repeat pairs of two sugars, d-glucuronic acid and *N*-acetyl-d-glucosamine (GlcNAc). HA sugar chains range between 15,000 and 32,500 disaccharide units long.

“It’s actually the most abundant nondietary sugar in our bodies, and there is plenty of it everywhere,” says Stavros Garantziotis, immunologist at the US National Institute of Environmental Health Sciences in North Carolina. “It’s deceptively simple in terms of how it’s built, but it’s very complex in what it does,” he says.

**Figure:**
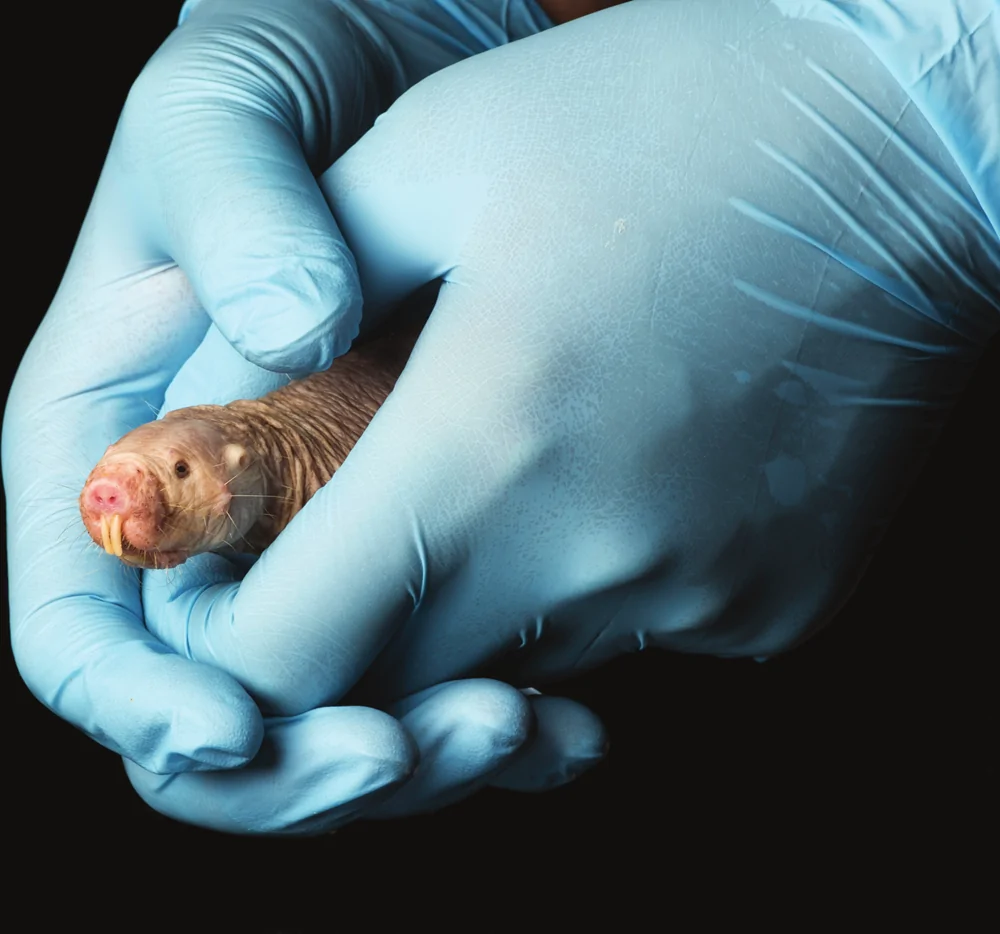
Naked mole rats evade cancer and other diseases thanks to the high levels of long-chain hyaluronic acid in their bodies. Credit: Vera Gorbunova.

HA’s main role is structural: it provides scaffolding, hydration, and lubrication because of how it binds water molecules. It creates a gelatinous polymer network that fills the extracellular matrix between cells. This quality allows it, for example, to act as a shock-absorber in the fluid that provides cushioning in bone joints.

But recent work from Gorbunova and others suggests it does much more. In 2023, Gorbunova’s laboratory group showed that some of the naked mole rat’s stunning health outcomes could be transferred to other rodents. The team lifted the genes that naked mole rats use to make exceptionally long HA chains and engineered that DNA into mice. The engineered mice had less cancer, lived 4.4% longer, and were healthier for longer than normal mice.

Sources: *Nature* 2013, DOI: 10.1038/nature12234; *Sci. Rep.* 2018, DOI: 10.1038/s41598-018-34445-0.

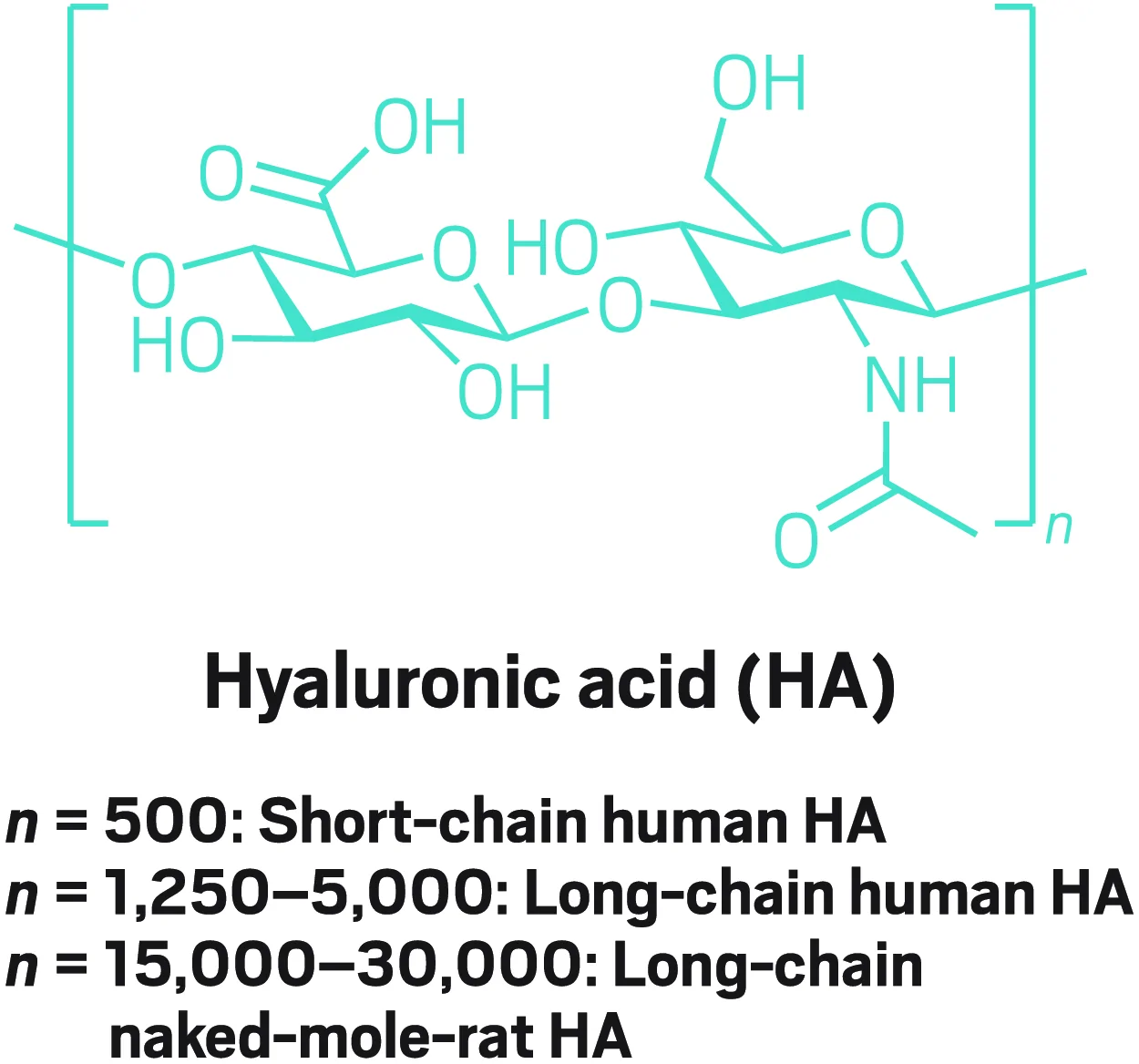

Gorbunova notes several mechanisms that explain HA’s effects. The large polymers create a physical barrier that stops cancer cells from migrating between tissues and metastasizing. Also, the mice with the naked mole rat gene showed less inflammation than other mice of the same age, Gorbunova says.

Long chains of HA are thought to reduce inflammation in several ways, including by directly regulating immune cell responses via the CD44 cell surface receptor. While shorter fragments cause a pro-inflammatory response via this receptor, the longer molecules are able to block that signaling cascade and stop the expression of inflammatory signals. Long-chain HA also forms cable-like structures that tangle up white blood cells and prevent them from triggering unnecessary immune responses.

Beyond understanding how long chains of HA behave differently from short ones, researchers are now probing the full life cycle of HA in the human body. They want to better understand the molecular machinery that creates—and breaks down—these long sugar chains and harness their effects for therapies.

## Making and breaking hyaluronic acid

In humans, HA is produced by hyaluronan synthases, the most studied being HAS2. This enzyme stitches together sugars and extrudes long-chain HA molecules to the outside of the cell.

Once HAS enzymes have meticulously built these chains, downstream enzymes called hyaluronidases break them down, cleaving the chemical bonds between sugar units. This breakdown happens in two stages. First, hyaluronidases outside the cell generate intermediate-length fragments. Next, those fragments enter the cell where another hyaluronidase chops them even smaller. These progressively shorter HA fragments—sometimes just a couple dozen sugars long—can accumulate and, paradoxically, start to cause damage.

Some shorter HA chains are necessary. For example, at wound sites, the body even uses one type of hyaluronan synthase that specifically generates shorter HA fragments. These fragments trigger an immune response that can protect the area. Plus the shorter chains are less of an obstacle to other immune cells and connective tissue cells that flood into the damaged area.

Problems start when the balance between making and breaking longer HA molecules becomes disrupted. Tumors can express high levels of hyaluronidases to break through tissue or to use HA as an energy source—it is made of sugars after all, says Paul Bollyky, an immunologist and clinician at Stanford University.

**Figure:**
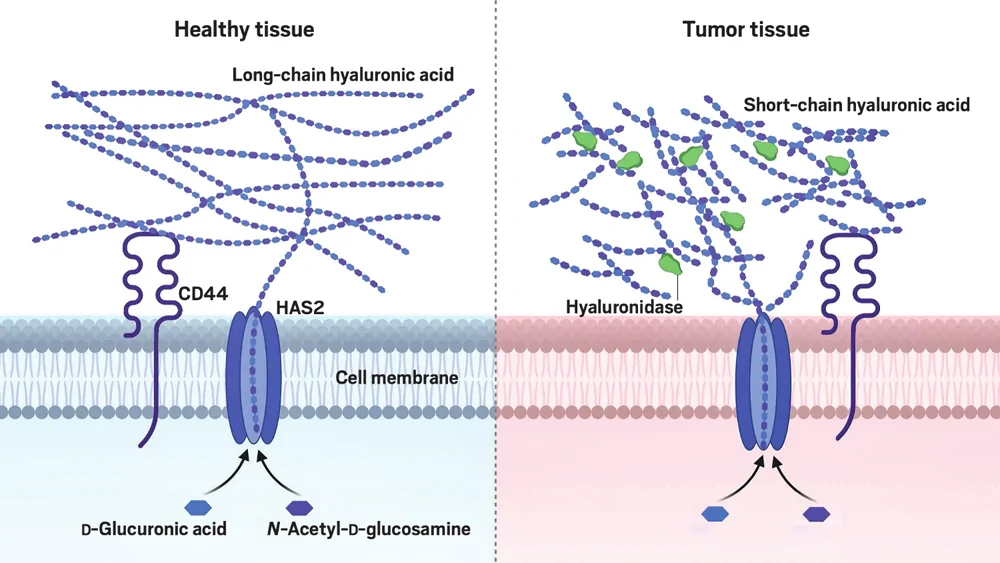
**Breaking the chain**. Long chains of hyaluronic acid (HA) are extruded outside of the cell by hyaluronan synthase enzymes such as HAS2. In healthy tissue (left), long chains of HA predominate and create a protective barrier around the cell. In cancerous tissue, tumors use hyaluronidase enzymes to break these chains down, allowing the cancer to move and metastasize. Short HA chains also activate receptors such as CD44, which induce inflammation and other harmful processes. Credit: Adapted from *Mol. Oncol.* 2023, DOI: 10.1002/1878-0261.13551.

The shorter HA fragments are also found in diseases associated with fibrosis, the thickening of connective tissue in response to injury or inflammation. When small HA fragments form but are not removed quickly enough, they keep trying to trigger an immune response. This process happens “in every organ that’s ever been studied,” says Garantziotis.

Bollyky has linked HA fragment accumulation to inflammation and fibrosis in several lung diseases as well. He thinks it may be implicated in autoimmunity and diabetes. Similar processes might even explain the gradual changes as we age. “Aging can be perceived as exposing yourself to some injurious stimulus again and again,” says Garantziotis.

## Hyaluronic acid as a therapy

HA is becoming the focus of therapeutic strategies for multiple diseases, and Gorbunova has returned to the naked mole rats for inspiration. While their humongous HA molecules are unlikely to have evolved specifically to give the animals longevity, Gorbunova suggests that their role is to protect these animals from reactive oxygen species in their low-oxygen, high–carbon dioxide underground homes.

In such hypoxic environments, metabolic reactions that depend on oxygen stop working as they should and can form harmful free radicals in the body. Longer HA chains help mop up those radicals by reacting with them and halting their progress. In effect, HA acts as a sacrificial barrier between the cell and molecules threatening it.

“Our approach has been to try to recreate a naked mole rat situation,” says Gorbunova. She has looked for a drug that could help retain more of the longer chains that human cells make and stop them from being fragmented.

To identify a drug candidate, her group carried out a high-throughput screen to find a molecule that could block the hyaluronidase. They identified delphinidin, a pigment found in fruits and vegetables, as a compound that boosts the level of long-chain HA in cell culture. When injected into mice, delphinidin reduced the migration and invasive behavior of breast, prostate, and melanoma cancer cells. Gorbunova’s group is now developing delphinidin as a natural supplement and looking for a more potent hyaluronidase inhibitor to develop as a cancer therapy.

Garantziotis has been trying a more straightforward approach: having patients inhale long chains of HA to alleviate certain inflammatory lung diseases. He wants to replace or supplement the natural supply of long-chain HA to try to “shove the short fragments of hyaluronan out of the way, which then produces less inflammation,” he explains.

Garantziotis and collaborators trialed the approach on patients with severe symptoms from chronic obstructive pulmonary disease (COPD) in intensive care. They found patients needed 1 day less of breathing support and were able to leave the hospital 3 days sooner.

Bollyky and others are focusing on reducing the overall amount of HA synthesized rather than trying to stop it being broken down. Although long chains of HA are necessary and even beneficial, in inflammatory disease the amount synthesized by cells is increased, and this volume contributes to the buildup of excess fragments when the long chains start to be broken down.

Bollyky’s team at Halo Biosciences, a start-up spun off from his lab, are developing a drug called hymecromone, also known as 4-methylumbelliferone (4-MU).

The compound blocks HA synthesis, via “a funny mechanism,” Bollyky says. To remove 4-MU from the body, the body first chemically tags it with the sugar glucuronic acid. This tagging is a common mechanism to make molecules more soluble and thus more easily excreted, he explains. But because glucuronic acid is also a precursor molecule to HA, 4-MU indirectly affects HA production. Without one of its feedstock molecules, the HA-producing enzyme HAS2 does not have much to do, and the body responds by turning down the enzyme’s expression.
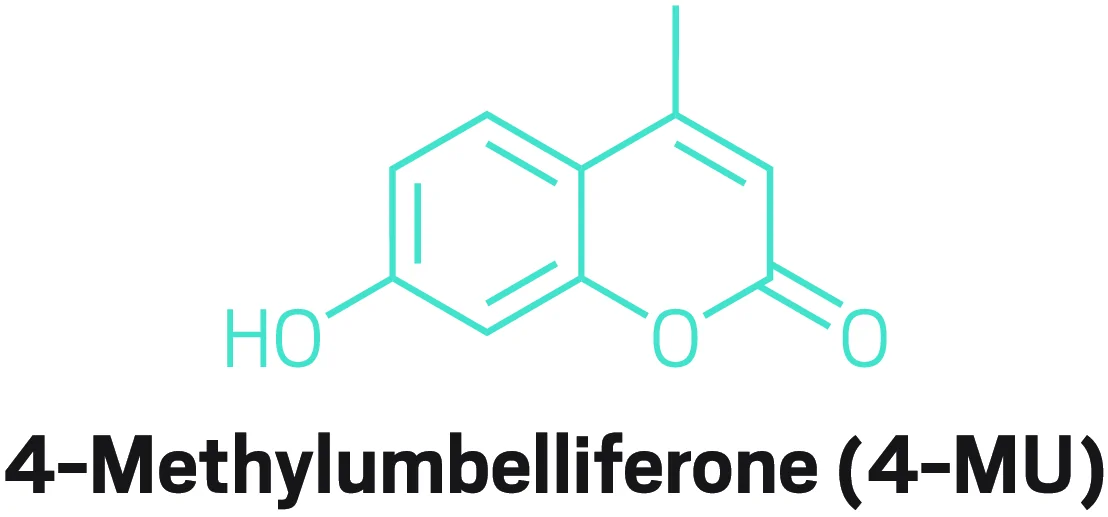


“You see less HA, and it tends to also shift the balance away from these low-molecular-weight fragments,” says Bollyky. Decreasing the production of HA altogether may seem counterintuitive, given that long chains of HA are protective. But slowing production allows for the small HA fragments that have built up to be completely broken down into their original, harmless sugar units.

Bollyky and his collaborators have had promising results studying the longevity of mice given 4-MU. In a 2024 paper, they showed that long-term administration of 4-MU extended median survival from 122 weeks in the control group to 154, with the maximum observed lifespan increasing from 159 to 194 weeks. “We could extend their lifespans by more than a year,” he says.

The drug is already approved in Europe and Asia to treat spasms from gallstones, but Halo is now testing it to treat patients with pulmonary hypertension associated with interstitial lung disease, a progressive illness that results from lung scarring. Its initial trial was successful in only a subset of patients, but Halo has embarked on a larger trial to see if their strategy can work.

While Bollyky’s team thinks HA is at play in 4-MU’s mechanism, there is still uncertainty about how the drug works. Earlier cell culture experiments by other research groups established a link between 4-MU and reduction in HA level, but Bollyky says his team’s results in mice do not show reductions in the HA levels of their tissue samples. That finding might be because the drug is causing only a small temporary inhibition of HA synthesis, he says, but that might be enough to shut down some of HA’s negative inflammatory effects.

Ekihiro Seki, an immunologist and liver specialist at Cedars-Sinai Medical Center, has been experimenting with 4-MU to treat liver disease. He found that certain liver cells overproduce HA, which ultimately leads to fibrosis and higher levels of HA fragments. His group then tested 4-MU in a mouse model of liver-disease-related fibrosis, and the drug lowered inflammation and fibrosis in those mice by suppressing HA synthesis.

But in 2025, the idea of developing 4-MU as a drug to treat liver diseases was challenged. A study from the lab of Yi Zhu at Baylor College of Medicine found that treating mice with 4-MU worked only at high doses that would likely be untranslatable to humans because of the drug’s toxicity.

4-MU may be useful in treating some diseases in which excess HA is produced, Gorbunova says, but for healthy mice and people, she thinks limiting the production of HA is likely to be detrimental. She says her team used 4-MU in some experiments on naked mole rats and noticed that “the animals start to look unhealthy.”

## Epigenetic approaches

An alternative strategy comes from biochemist Davide Vigetti at the University of Insubria. He has studied how HA plays a part in atherosclerosis, the process that causes arteries to become clogged by fatty plaques that can ultimately lead to ruptures or blockages.

Vigetti is looking at some of the epigenetic ways that HA production and breakdown are controlled in that process. In particular, he’s homing in on how the HAS2 enzyme is up- or downregulated.

He discovered a long noncoding RNA hundreds of base pairs long made from the sequence opposite to that of the *HAS2* gene. The RNA’s role is to bind to the *HAS2* gene, keeping the DNA double helix open and amplifying transcription of *HAS2*. In turn, this intervention could create more HA.

RNA can also work in the reverse direction to lower HA production. Seki has explored the role of a microRNA called miR-200c. The 22-nucleotide sequence works in the cell cytoplasm and binds to the tail end of the mRNA transcript that codes for HAS2. That tiny interaction is enough to prevent HAS2 from being produced. Less HAS2 would lead to less HA and could potentially reduce harmful HA fragments—the same rationale being used for treatment with 4-MU.

So far these epigenetic approaches have not been translated into therapies, but they present yet another avenue for ‘manipulating’ the complex balancing act of HA.

These efforts to control HA levels stem largely from Gorbunova’s decades of studying the naked mole rat. Now chemists might be on the cusp of harnessing some of the rodent’s powers for human benefit. “When we came in, we just wanted to understand the secret of the cancer resistance,” Gorbunova says. “It’s amazing to see what we’ve learned.”

*Rachel Brazil is a freelance contributor to* Chemical & Engineering News *, the independent news outlet of the American Chemical Society*.

